# What Does Cleft Lip and Palate Care Cost? The Time and Economic-Associated Burden of Care From Birth to Maturity

**DOI:** 10.1177/22925503231203216

**Published:** 2023-10-09

**Authors:** Emma Wells-Durand, Angela Buchel, Young Ji Tuen, Richard Thomson, John Staples, Travis L Gibson, Angelina Y C Loo, Brian McClung, Sheryl Palm, Jugpal S. Arneja

**Affiliations:** 1 Division of Plastic Surgery, Department of Surgery, 8166 University of British Columbia, Vancouver, British Columbia, Canada; 2 Department of Oral Health Sciences, 8166 University of British Columbia, Vancouver, British Columbia, Canada; 3 Data Analytics, Reporting and Evaluation, 8145 Provincial Health Services Authority, Vancouver, British Columbia, Canada; 4 Department of Audiology & Speech, 8166 University of British Columbia, Vancouver, BC, Canada; 5 BC Children's Hospital Research Institute, Vancouver, British Columbia, Canada; 6 Sauder School of Business, 8166 University of British Columbia, Vancouver, British Columbia, Canada

**Keywords:** cleft lip and palate, costs and cost analysis, burden of care, child, fente labiale et palatine, coûts et analyse des coûts, fardeau des soins, enfant

## Abstract

**Introduction:** This study aims to describe the burden of care (BoC) for the management of patients with nonsyndromic cleft lip and palate (CLP) by identifying provider burden, characterizing an interaction burden, and calculating an economic burden associated with their health system interactions. **Methods:** A retrospective chart review was conducted of patients with nonsyndromic CLP treated at a pediatric tertiary hospital between January 1, 1999, and April 30, 2021. Healthcare utilization data for inpatient and outpatient interactions were extracted. Community outpatient data were obtained from affiliated specialists. Bottom-up microcosting was utilized for hospital costing, the provincial tariff guide for provider reimbursement, and zip code for calculating patient costs. **Results:** In total, 58 patients identified with CLP had a median of 148.5 healthcare interactions (consults/follow-ups/surgeries) between the ages of 0 and 18 years. Patients had a median of 10.5 surgical procedures, and a median 135.8 outpatient interactions. The most used specialty service was orthodontics, with a median of 71.5 orthodontic interactions per patient. The median cost of care, including direct hospital costs, physician costs, community healthcare costs, and indirect costs, was $73,398. **Conclusions:** Patients born with nonsyndromic CLP have a very high frequency of healthcare encounters, out of proportion to cost associated with healthcare, suggesting an overall significant BoC.

## Résumé

**Introduction :** Cette étude vise à décrire le fardeau des soins que représente la gestion des patients atteints d’une fente labiale et palatine (FLP) non syndromique en identifiant le fardeau pour le prestataire de soins, définissant le fardeau des interactions et calculant le fardeau économique associé à leurs interactions avec le système de santé. **Méthodes :** Il s’agit d’une revue rétrospective des dossiers de patients atteints de FLP non syndromique traités dans un hôpital pédiatrique de niveau 3 entre le 1er janvier 1999 et le 30 avril 2021. Les données d’utilisation du système de santé pour les interactions en tant que patients hospitalisés et ambulatoires ont été extraites. Des données pour des patients ambulatoires traités dans la communauté ont été obtenues auprès des spécialistes affiliées. Un calcul ascendant des microcoûts a été utilisé pour les coûts hospitaliers, le guide des tarifs provinciaux pour le remboursement des prestataires de soins et le code postal pour le calcul des coûts incombant aux patients. **Résultats :** En tout, 58 patients atteints de FLP ont été identifiés, avec une médiane de 148.5 interactions avec le système de soins (consultations, suivis, chirurgies) de la naissance à l’âge de 18 ans. Les patients ont subi un nombre médian de 10,5 procédures chirurgicales et un nombre médian de 135.8 interactions en tant que patients ambulatoires. Le service spécialisé le plus utilisé était l’orthodontie avec un nombre médian de 71,5 interactions orthodontiques par patient. Le coût médian des soins, incluant les coûts hospitaliers directs, les honoraires de médecins, les coûts de soins communautaires et les coûts indirects, a été de 73 398 dollars. **Conclusions :** Les patients nés avec une FLP non syndromique connaissent une grande fréquence de rencontres avec le système de soins et des coûts substantiels associés aux soins, suggérant une fardeau des soins majeure.

## Introduction

### Cleft Lip and Palate

Orofacial clefts (OFCs) are the most common congenital craniofacial malformation, affecting approximately 1 in 700 births,^1^ and the second most common congenital anomaly.^2^ Management of cleft lip and palate (CLP) is complex and requires a multidisciplinary approach to care, which can include nursing, pediatrics, plastic surgery, oral surgery, anesthesiology, otolaryngology, speech-language pathology (SLP), audiology, social work, psychology, genetics, orthodontics, prosthodontics, and dentistry to achieve functional and aesthetic results.^3-5^ Although rehabilitation is possible with a team-oriented approach to care, the management of OFCs such as CLP places an inevitable burden on the patient and their family,^3^ resulting in significant expenditure by the patient and healthcare system.^6^

### Burden of Care

Burden of care (BoC) refers to the total cost of being a patient, including direct monetary costs, indirect costs, and total hospital and healthcare service use.^7^ The BoC for patients with CLP includes the total number of inpatient and outpatient visits throughout the entire duration of treatment; the full financial burden of treatment for the patient and hospital; and the indirect burden of travel, hotel costs, and missed workdays associated with obtaining treatment.

### Gaps in Knowledge

Despite anecdotal evidence suggesting that patients with CLP have a high frequency of encounters with the healthcare system, there is currently a lack of Canadian literature demonstrating so. Historically, the concept of BoC in CLP care has narrowly focused on inpatient and outpatient care, financial cost, and travel time.

Existing research on inpatient care utilization for CLP investigates the type and frequency of hospital admissions.^8-11^ While there is much research investigating the inpatient BoC as it relates to surgical, orthodontic, and nasoalveolar procedures,^9,10,12-16^ minimal research investigates the BoC relating to outpatient visits, such as those in the specialist disciplines of antenatal care, general pediatrics, otolaryngology, SLP, audiology, psychology, social work, and genetics. One well-studied facet of outpatient CLP care is orthodontics. However, data are currently lacking on the frequency and cost of most other outpatient visits, and how they contribute to overall BoC.

The Canadian cost of CLP care remains understudied. Because most microcosting and economic analyses have been conducted in countries with healthcare systems different from Canada's, existing data may have limited applicability to the Canadian system. To our knowledge, the indirect financial BoC for CLP families, including expenses related to travel, accommodation, parking, and job absenteeism,^17^ has not been studied in the context of families in British Columbia (B.C.), Canada. Moreover, significant distance barriers may further increase healthcare utilization and raise direct costs, as doctors may prolong postoperative hospital stays to avoid problematic return travel.

### Scientific Justification

To the best of our knowledge, there is currently no comprehensive review investigating the complete BoC, including a utilization and economic analysis of nonsyndromic CLP treatment from birth to maturity. The present study aims to (1) identify and characterize the healthcare interactions of pediatric patients with nonsyndromic CLP in B.C. and (2) calculate the patient, provider, and system financial costs related to such healthcare interactions. Our results hope to inform patient counseling regarding BoC associated with a CLP diagnosis, improve the understanding of costs associated with a CLP diagnosis, and motivate the development of more efficient healthcare systems that maximize patient access to care.

## Methods

This study is a retrospective chart review from January 1, 1999, to April 30, 2021, approved by the University of British Columbia Children's and Women's Health Centre of British Columbia Research Ethics Board (#H21-01293). We defined the BoC for CLP as a combination of the healthcare utilization burden and the financial burden faced by patients, providers, and the healthcare system itself. Utilization was characterized by the care level, frequency, and provider specialty service of each healthcare interaction. Financial burden was defined as the sum of indirect patient, healthcare provider, and hospital costs associated with each healthcare interaction.

### Patient Population

Our hospital is the only quaternary pediatric hospital providing specialized CLP care in the province. Its CLP clinic receives all children in B.C. who require specialized, team-oriented CLP care, and currently serves approximately 1800 active patients. The patients we studied were B.C. residents diagnosed with CLP (unilateral or bilateral, complete or incomplete) who had completed their pediatric care and were born between January 1, 1999, and April 30, 2003. We excluded patients with known syndromes, those who were surgically treated outside of our hospital, those with incomplete health records, and those with cleft lip only (CLO) or cleft palate only (CPO).

### Healthcare Utilization

A healthcare encounter was defined as a single scheduled interaction with a specialist care provider, either in an outpatient or surgical setting. All healthcare encounters for each patient were recorded, including details such as age at interaction, care level (surgical [surgical day care and inpatient]/outpatient/emergency), primary specialty service, type (procedure/consult/follow-up), and duration (length of stay). Healthcare encounter data were collected from different sources according to the date each source was established, including electronic hospital databases and paper charts.

Complete data on oral surgery, SLP outpatient encounters, and orthodontic encounters were not available for our patient population due to difficulty accessing data from privatized clinics. Thus, oral surgery encounter averages were estimated by an affiliated oral surgeon according to the number and type of oral surgeries each patient underwent. SLP encounters were estimated by an affiliated speech-language pathologist for a random 19-patient subset of our study population, from which medians were calculated and extrapolated to the remaining 39 patients. Outpatient orthodontic encounters were also not available for 38 patients. Therefore, true orthodontic healthcare encounters occurring in the community were collected from an affiliated clinic for a random 20-patient subset of our study population, from which medians were calculated and extrapolated to the remaining 38 patients.

### Microcosting Approach

#### Hospital costs

Cost-per-minute or cost-per-activity rates were generated and multiplied against the activity consumed by each patient to calculate the total hospital cost. Physician costs were calculated separately and reported as provider costs, as described in the next section.

Weighted operating room (OR) minutes were used to calculate OR costs, and are equal to the number of staff present at each surgery multiplied by the length of the surgery in minutes. Calculated OR costs account for nursing staff, clerks, and supplies, but not for physicians, perfusionists, or anesthesia technicians.

Hospital outpatient clinic costs were calculated by dividing the total number of outpatient visits for each clinic by the assigned expenses to generate a cost-per-visit. Due to an incomplete dataset, it was not feasible to generate a cost-per-minute rate for allied health workers (physiotherapists, occupational therapists, clinical nutritionists, psychologists, and social workers); therefore, we substituted a cost-per-minute rate based on the average salary of each service.

#### Provider costs

Provider costing was defined as the total cost paid to healthcare providers involved in the CLP care of our patient population. Of note, costs for nurses, imaging technicians, physiotherapists, occupational therapists, clinical nutritionists, psychologists, and social workers at our hospital were already included in hospital costs.

Surgical and outpatient physician costs were collected from the 2021 provincial fee-for-service assessment and procedural guide. Nonphysician provider costs were based on specialist suggestions from hospital-affiliated providers in audiology, orthodontics, and SLP. Private community orthodontic fees were collected as total care cost ledgers. SLP and private community orthodontic provider costing rates were applied to random 19- and 20-patient subsets of our study population, respectively, after which medians were extrapolated and applied to the remaining 39 and 38 patients, respectively.

#### Indirect patient costs

Indirect BoC was defined as patient costs of travel, parking, accommodation, and missed caregiver workdays as a result of healthcare interactions occurring at our hospital. Travel costs were estimated as the distance between our hospital and patient postal codes at the time of each encounter multiplied by the 2020 gas metric of $0.53/km.^18^ A parking rate of $14/day^19^ was applied to healthcare interactions occurring on unique days.

Patients residing a significant distance away from our hospital and who were attending surgical or outpatient encounters with 2 or more specialists were assumed to have stayed overnight as multidisciplinary visits at the cleft palate clinic which often begin at 8 am. Hence, accommodation costs of $100/night, based on the average 1 night stay at the Ronald McDonald House,^20^ were applied to encounters of patients residing at least 50 km away from our hospital.

Caregiver's wage (absenteeism) was estimated by applying the median daily wage with benefits for individuals 15 years and over ($189.13)^21^ to hospital length of stay and number of outpatient visits.

### Statistical Analysis

Descriptive statistics characterized inpatient, surgical day care, outpatient, and emergency healthcare utilization data, as well as all aspects of cost. In our analysis, we included SLP and community orthodontic encounters in our healthcare utilization data estimates. All costs were adjusted to the 2019/2020 fiscal year to account for inflation.

## Results

A total of 58 eligible participants who met our inclusion criteria were identified from our hospital's Cleft Palate-Craniofacial Clinic Database.

### Healthcare Utilization

#### Overall utilization

**Figure 1.**
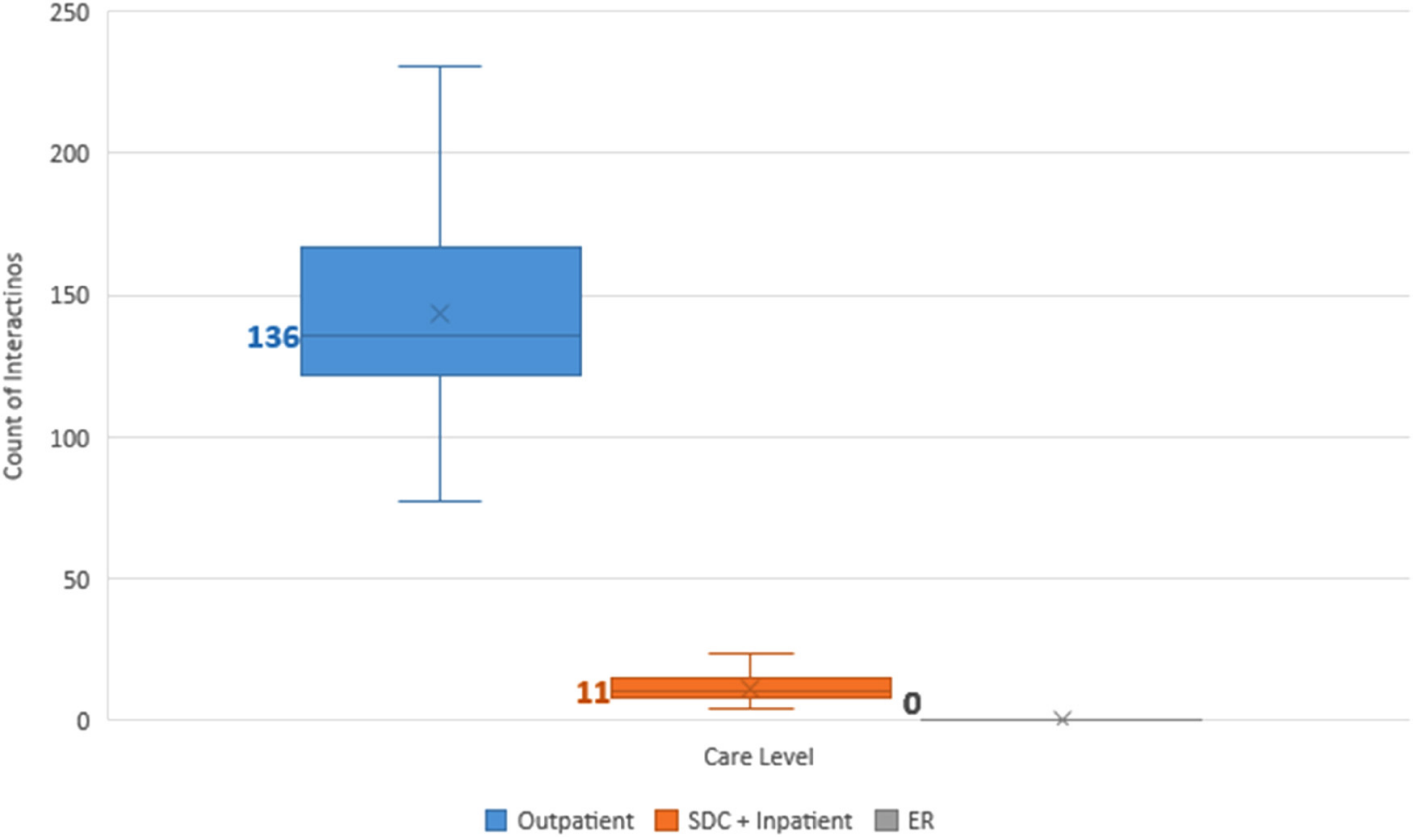
Median number of interactions with the healthcare system experienced by children born with cleft lip and palate (CLP) in B.C., sorted by care level (outpatient, surgical day care [SDC] and inpatient, or emergency room [ER]). Mean values are represented as crosses, and median values are in bold and denoted as lines.

Our study population had a median of 148.5 interactions (range 57-298) with the healthcare system before maturity. Of these, a median of 93% were outpatient encounters, 7% were surgical, and 0% were emergency room encounters (Figure 1).

#### Surgical interactions

**Figure 2.**
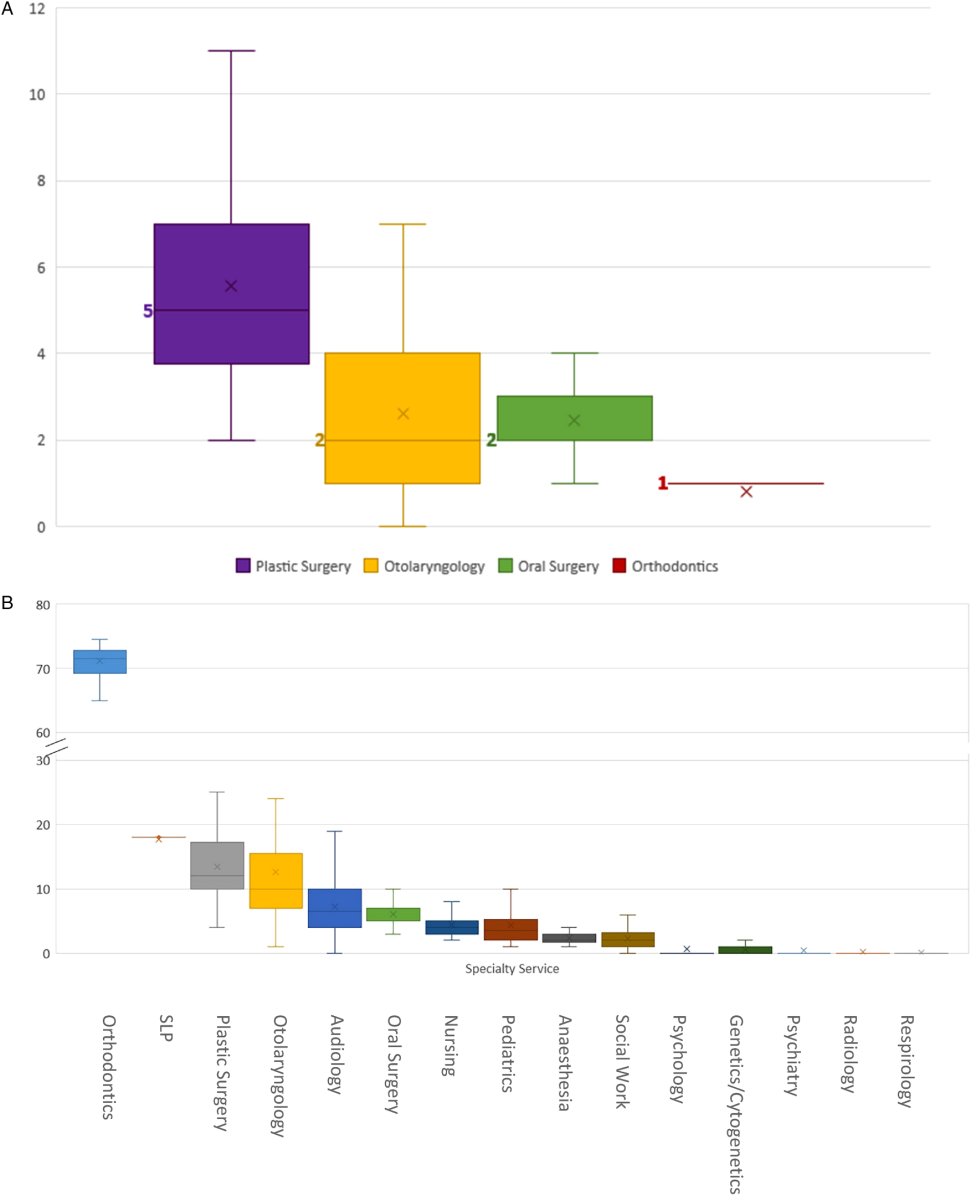
(A) Median number of surgical interactions (including surgical day care and inpatient stays) with the healthcare system experienced by children born with cleft lip and palate (CLP) in B.C., sorted by physician specialty service. (B) Median values are in bold and denoted as lines. B, Median number of outpatient interactions with the healthcare system experienced by children born with CLP in B.C., sorted by provider specialty service. Median values are denoted as lines.

The 58 patients had a median of 10.5 surgical interactions (range 4-24), including inpatient (median 6.5) and surgical day care (median 3) encounters (Figure 1). Surgical specialty services encountered, in order of descending medians, were plastic surgery (5), otolaryngology (2), oral surgery (2), and orthodontics (1) (Figure 2A).

#### Outpatient interactions

**Figure 3.**
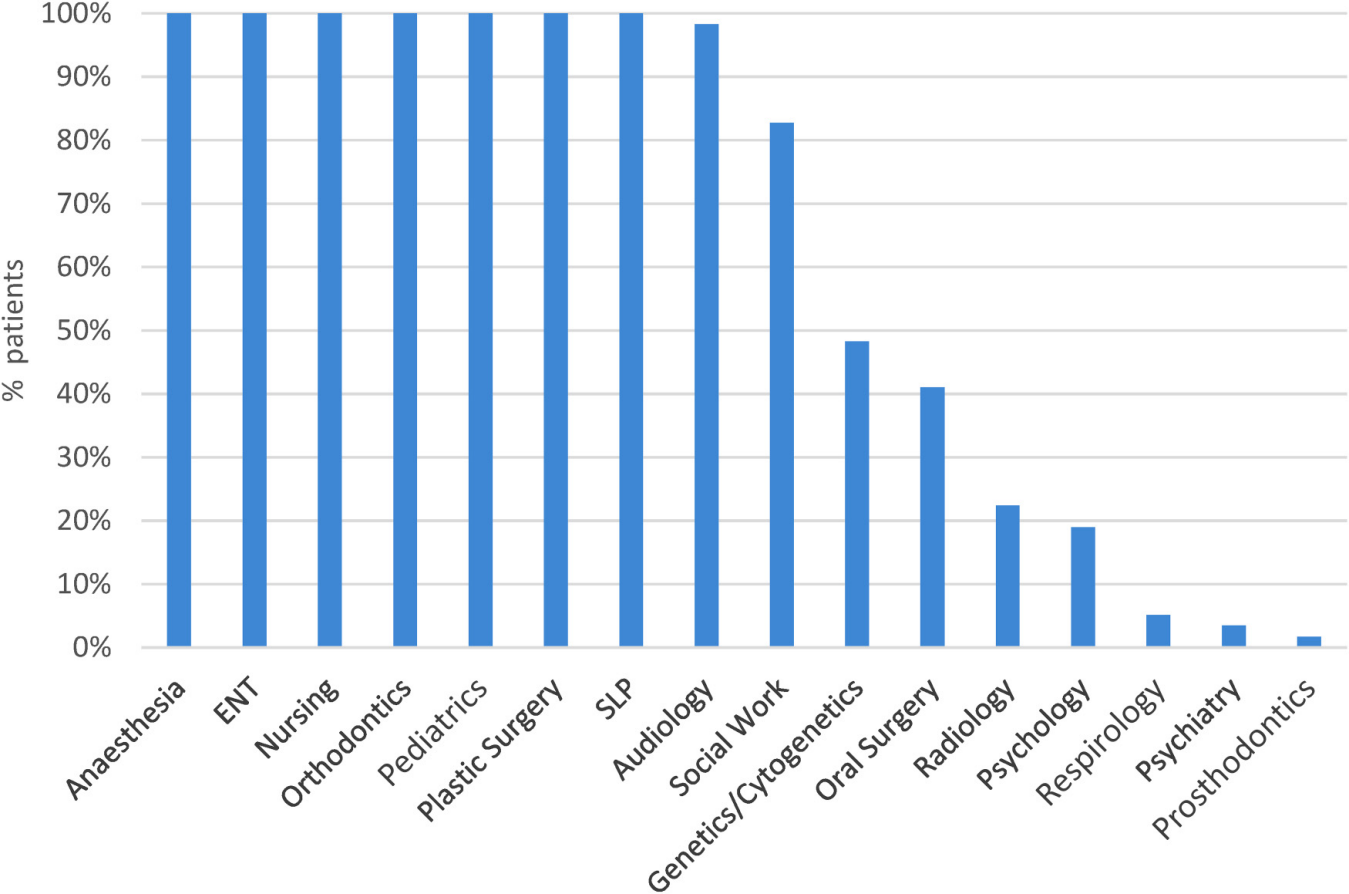
Percent of children born with cleft lip and palate (CLP) who interacted with specialty services.

Our study patients had a median of 135.8 outpatient interactions (range 50-274) with the healthcare system (Figure 1). Outpatient specialty services encountered, in order of descending medians, were orthodontics (71.5), SLP (18), plastic surgery (12), otolaryngology (10), audiology (6.5), oral surgery (5), nursing (4), pediatrics (3.5), anesthesia (2), social work (2), psychology (0), genetics (0), psychiatry (0), radiology (0), and respirology (0) (Figure 2B). Figure 3 depicts the proportion of patients who interacted with different outpatient specialties. Of note, 100% of patients interacted with specialists of the following disciplines in outpatient settings: anesthesia, otolaryngology, nursing, orthodontics, pediatrics, plastic surgery, and SLP (Figure 3).

#### Emergency room interactions

The study population had a median of 0 emergency room interactions (range 0-6) (Figure 1).

### Cost

**Figure 4.**
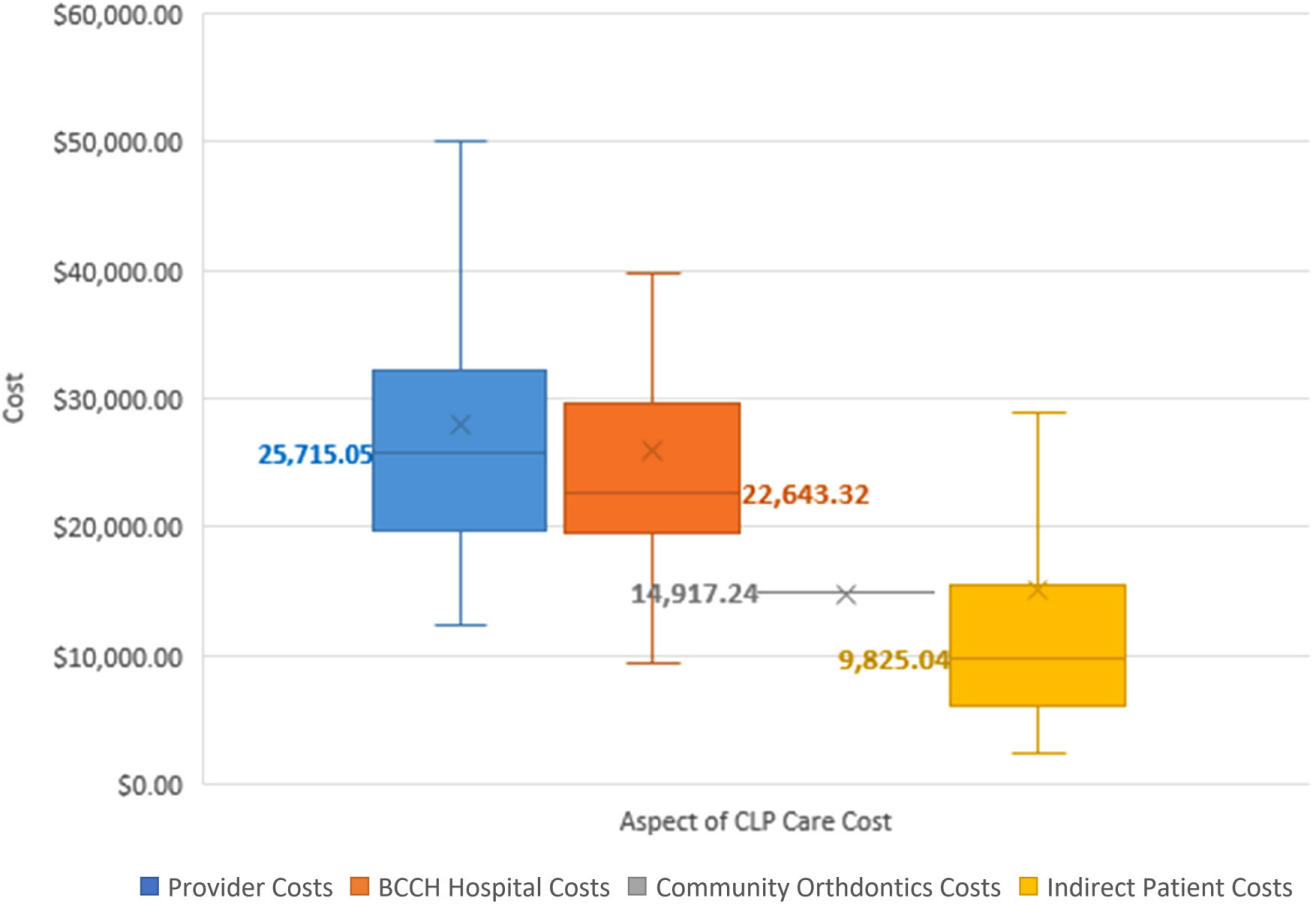
Median costs associated with cleft lip and palate (CLP) care from birth until maturity in B.C. Median values are in bold and denoted as lines. Outlier values are not included.

**Figure 5.**
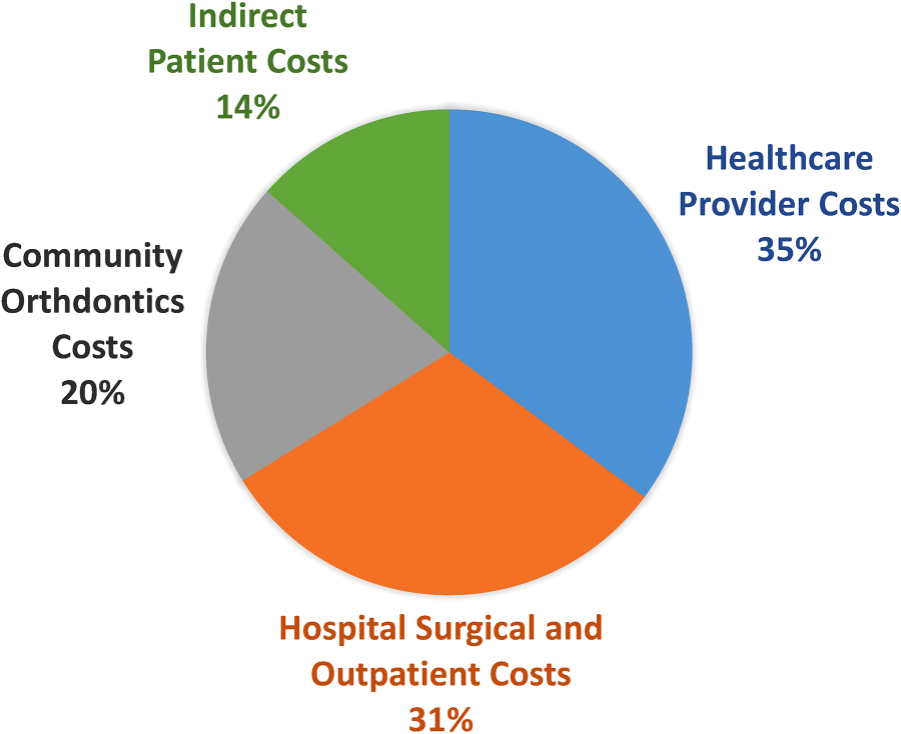
Median percent of cost allocation of cleft lip and palate (CLP) care from birth to maturity in B.C.

The total cost of CLP care from birth to maturity in our province was calculated to be a median of $73,398 (range $45,874-$292,689). Aspects of care, in order of descending cost medians, were healthcare provider costs ($25,715), hospital surgical and outpatient costs ($22,643), community orthodontic costs ($14,917), and indirect patient costs ($9825) (Figures 4 and 5). Surgical physician costs (median $14,376), outpatient provider costs (median $8313), and emergency physician costs (median $0) comprise the provider costs described in Figures 4 and 5.

## Discussion

Our study population of 58 patients with CLP had a median of 148.5 interactions with the healthcare system, including a median of 10.5 surgeries, costing a median of $73,398 to the healthcare system and to the families.

### Healthcare Utilization

#### Surgical care

Hospital admissions are a significant component of the BoC for CLP, in part due to their resource-intensiveand costly nature. While the present study focuses on children with CLP in B.C., many studies from other areas of the world have compared hospital admissions between children with and without oro-facial clefts (OFCs), as well as between children with different types of OFCs. Australian children born with OFCs of any type were 3 times more likely to be admitted to hospital and had a greater number of admissions compared to children without OFCs.^8^ As expected, patients with CLP were admitted to hospital for cleft-related reasons at a higher rate in every age group than were those with cleft lip only (CLO) or cleft palate only (CPO).^8^ A different Australian study determined that hospitalization rates were 4 times higher for patients living in high-access regions.^10^ While the present study did not report differences in admission rates according to geographic proximity to CLP care, this literature is relevantfor comparison with B.C., where a significant percentage of the province does not have close access to care at our hospital.

The type and frequency of hospital procedures for the management of CLP have only been studied to a minimal extent outside of the present study, despite having significant implications for the BoC experienced by patients. We discovered that patients with CLP undergo surgical procedures with a median of 10.5 times before maturity, similar to the median of 11 hospital admissions occurring before maturity reported by Bell et al.^8^ In contrast, a Norwegian 20-year follow-up of patients with complete unilateral CLP found that patients underwent a lower mean of 4.8 surgeries before age 20.^9^ Likewise, Schnitt et al^11^ reported a mean of 5.7 surgical interventions during the duration of unilateral CLP care. Our findings indicate that plastic surgery was the most encountered surgical specialty service, followed by otolaryngology, oral surgery, and lastly orthodontics. The frequency of inpatient procedures undergone by patients with CLP is an important contributor to BoC, due to its influence on the indirect BoC for families, and overall cost to the healthcare system. Increased procedure frequency yields a longer total length of stay in hospital, and therefore increased demands for commuting families, further explored in our discussion of *Indirect Patient Costs*.

#### Outpatient care

Understanding the type and frequency of outpatient encounters in CLP care is critical to understanding our patients’ BoC, but made difficult by the scarcity of available and relevant literature. Our study investigated the varieties of outpatient care encountered by patients with CLP, reporting a median of 135.8 interactions with outpatient care services. We found that the 5 most encountered outpatient specialty services in descending order were orthodontics, SLP, plastic surgery, otolaryngology, and audiology. While existing literature does not reveal a comprehensive account of outpatient interactions for comparison, some data exist for individual aspects of outpatient care. For example, Trivedi et al^22^ found the percentage of patients with CLO and CPO either receiving or being recommended for SLP and child psychotherapy to be 64% and 26%, respectively.^22^ Our study reports more prevalent SLP engagement at 100% of patients, likely attributable to the more extensive nature of a CLP diagnosis, and comparable psychology engagement at 19% of patients. In a broad analysis of healthcare utilization for patients with OFCs, Pedersen et al^23^ reported the greatest specialty service usage in patients with CLP, compared to those with other OFCs—a comparison not explored in the present study. They reported an average use of 5.7 physician services,^23^ in contrast to our finding that 97% of patients encounter at least 9 specialty services over the course of their CLP care. It is unclear whether our definition of “specialty service” captured more services, possibly explaining the greater number of specialists encountered by our study population. It should be noted that our reported range for SLP and orthodontic care is small, due to a limitation in our data collection process; gaps in SLP and orthodontic total encounter numbers were filled with median values derived from smaller sample sizes. Unfortunately, comprehensive data on the number of CLP encounters with specific outpatient services such as plastic surgery, SLP, otolaryngology, audiology, oral surgery, nursing, pediatrics, anesthesia, social work, psychology, genetics, radiology, and respirology are not yet available, to our knowledge.

One well-studied area of outpatient healthcare utilization in the context of CLP management is orthodontics. One Australian analysis examining Medicare claims for CLO- and CPO-related outpatient services discovered that orthodontic services comprised the majority of claims (70.1%), followed by prosthodontic services (22.5%) and oral surgery services (7.5%).^10^ This trend is in keeping with our results, where orthodontic services make up a 53% majority of outpatient healthcare interactions, while oral surgery services comprise 4%. Similar median numbers of outpatient orthodontic interactions to our study have been reported by Polish and Brazilian studies; we report a median of 71.5 orthodontic encounters while they both report a median of 62.^14, 16^ In contrast, Hameed et al^13^ report a slightly lower average of 44 orthodontic appointments for complete orthodontic management of any OFC. We suspect their comparably lower average to represent the management of less complex diagnoses such as CLO and CPO. While the vast majority of outpatient healthcare utilization for CLP care remains understudied, research has established that a universally significant BoC for orthodontic CLP management exists.^24^

### Cost

#### Provider and hospital costs

Our findings indicate that providercosts are the most expensive aspect of BoC, followed by hospital facility costs, and lastly indirect patient costs. While Canadian research examining the cost breakdown of CLP care is lacking, studies from other countries illustrate a significant cost burden. A retrospective study investigating healthcare use of American cleft patients provides a rich background on cost burden and indicates that the average total cost of CLP care at 18 months of age is $35,826.^25^ Overall, 79% of the total cost for healthcare services was attributed to inpatient visits, and 21% to unspecified outpatient visits.^25^ This, in conjunction with prior reports that over half of all hospital admissions occur in the first year of life,^12^ may allow us to approximate the total cost of care at maturity as roughly double that reported at age 1.^25^ If so, this figure would be comparable to our reported median total cost of $73,398. Their microcosting analysis attributed 55.1% ($19,818) of the total cost to hospital charges, 33.8% ($12,103) to physician charges, and 10.9% ($4031) to patient charges.^25^ This is in contrast to the present study, where hospital costs comprised only 31% of the total cost. While we report a similar proportion of provider costs (35%), a notable difference in our study is the inclusion of allied health workers in our provider cost calculation. It should also be noted that our provider cost calculation did *not* include the cost of private orthodontic providers and supplies. Thus, our separately reported provider costs may appear underrepresented.

Despite their comprehensive microcosting analysis, the American study^25^ examines a time frame of only 18 months. When the study time frame is expanded to the first 4 years of life as was done by Ligh et al,^26^ the average total cost of CLP care increases to $91,263. Indeed, most studies that examine cost beyond 18 months have found total costs of CLP care to be within $65,000 and $100,000^27,28^ and indicate that children with CLP have hospital charges approximately 8 times greater than that of children without CLP.^24,29^ Although no studies examine total cost to maturity, past literature consistently reveals that inpatient healthcare expenditure decreases with increasing age, with the highest financial cost occurring within the first 2 years of treatment.^24,27,30^ Therefore, costs provided by studies with shortened timeframes likely underestimate the total cost of CLP care, though not to an overwhelming extent, as CLP care is front-loaded.

#### Indirect patient costs

The direct use of hospital and clinic resources is not the sole financial cost of CLP care. Many indirect burdens exist, including travel time and distance to CLP care, overnight hotel stays, and missed workdays or “absenteeism.” We calculated the median indirect BoC, including travel, accommodation, parking, and absenteeism costs to be $9825. Indirect costs may be particularly significant in our province, where families living further away must travel longer distances to receive care. Recently, Leung et al^31^ noted that in 2016, only 62% of our hospital's cleft program's patient population lived in the greater Vancouver, B.C. area. Furthermore, while 38% of the study population regularly traveled from distant locations within B.C., an additional 4 patients did so from the Yukon.^31^ Such greater distances not only exacerbate the indirect BoC by way of adding transportation costs, missed workdays, and accommodation fees but could also introduce potential for more frequently missed appointments and subsequently worse outcomes.

### Limitations

The present study may be limited in that we did not investigate indirect BoC variables including lost parental career opportunity, caregiver burnout, missed school days, child psychosocial costs, and overall quality of life. Such indirect factors may contribute to an overall greater qualitative BoC from the patient perspective than is reported in our strictly quantitative results. In addition, our dataset is incomplete in certain domains of outpatient care including outpatient SLP, outpatient oral surgery, and outpatient orthodontics. Future studies may consider investigating the qualitative BoC faced by patients and length of stay as it pertains to the indirect and cost BoC. Future research may also consider comparing the Canadian BoC for different cleft presentations, including CLO, CPO, and CLP.

## Conclusion

Cleft lip and palate is a congenital malformation for which care in B.C. is multidisciplinary and based largely at 1 hospital, for a provincial population of about 5 million. The term “*burden of care*” describes the time-related and financial costs of being a patient, including the extent of healthcare utilization as well as direct and indirect costs. Anecdotal evidence suggests that patients with CLP experience a significant BoC associated with their diagnosis, which has been confirmed for individual aspects of care by several subspecialty groups. However, there has been no comprehensive investigation of the full BoC, including a healthcare utilization and Canadian economic analysis to date. The present study is a retrospective chart review identifying and characterizing the frequency, type, and cost of CLP healthcare interactions in B.C. from birth until age 18. We found that children have a median of 148.5 interactions with the healthcare system, composed of a median of 10.5 surgical and 135.8 outpatient encounters. The median total cost of care, including hospital, provider, and indirect patient costs, was $73,398. Our research hopes to influence providers by providing data to optimize clinic capacity and efficiency, and patients by informing what BoC families and caregivers may experience regarding a CLP diagnosis, and finally the administrators and payors in our healthcare system through attaching a case cost. Patients born with nonsyndromic CLP have a very high frequency of healthcare encounters, out of proportion to cost associated with healthcare, suggesting an overall significant BoC. We suggest that characterizing the costs and BoC for CLP is critical in improving patient care, right-sizing access, and managing the expectations associated with care.

## Supplemental Material

**Figure:** Video 1.
